# Over-Expression of Rose *RrLAZY1* Negatively Regulates the Branch Angle of Transgenic Arabidopsis Inflorescence

**DOI:** 10.3390/ijms222413664

**Published:** 2021-12-20

**Authors:** Dan Li, Mingyuan Zhao, Xiaoyan Yu, Lanyong Zhao, Zongda Xu, Xu Han

**Affiliations:** College of Forestry, Shandong Agricultural University, Taian 271018, China; lidan6108@163.com (D.L.); zhaomingy9@163.com (M.Z.); yxyxst20040214@163.com (X.Y.); sdzly369@163.com (L.Z.)

**Keywords:** *RrLAZY1*, branch angle, *Rosa rugosa*, over-expression, protein interaction

## Abstract

Branch angle is a key shoot architecture trait that strongly influences the ornamental and economic value of garden plants. However, the mechanism underlying the control of branch angle, an important aspect of tree architecture, is far from clear in roses. In the present study, we isolated the *RrLAZY1* gene from the stems of *Rosa rugosa* ‘Zilong wochi’. Sequence analysis showed that the encoded RrLAZY1 protein contained a conserved GΦL (A/T) IGT domain, which belongs to the IGT family. Quantitative real-time PCR (qRT-PCR) analyses revealed that *RrLAZY1* was expressed in all tissues and that expression was highest in the stem. The RrLAZY1 protein was localized in the plasma membrane. Based on a yeast two-hybrid assay and bimolecular fluorescence complementation experiments, the RrLAZY1 protein was found to interact with auxin-related proteins RrIAA16. The over-expression of the *RrLAZY1* gene displayed a smaller branch angle in transgenic Arabidopsis inflorescence and resulted in changes in the expression level of genes related to auxin polar transport and signal transduction pathways. This study represents the first systematic analysis of the *LAZY1* gene family in *R. rugosa*. The results of this study will provide a theoretical basis for the improvement of rose plant types and molecular breeding and provide valuable information for studying the regulation mechanism of branch angle in other woody plants.

## 1. Introduction

*Rosa rugosa*, belonging to the Rosaceae family, has showy flowers and a strong fragrance [1]. It is an important spring flower species in garden landscaping. In recent years, domestic and foreign research on roses has mainly focused on flower color, floral fragrance, breeding, variety classification, and flowering period regulation, and the study of the plant architecture has been very limited [1,2,3,4]. Plant architecture is an important ornamental element of garden plants and plays a vital role in the composition of garden landscapes. At present, most of the existing rose varieties in China are erect, appearing in the form of shrubs. Thus, single-plant architecture is the limiting condition for the further popularization of rose plants in gardens [5]. Therefore, the study of rose creeping plants will help to increase the application of rose plants and is of great significance in creating more diverse garden plant types.

Tiller angle is an important component of plant architecture. In addition to environmental and hormonal factors, the branch angle is regulated by specific genes [6]. In recent decades, several genes affecting rice tiller angle have been identified. Tiller angle control 1 (*TAC1*) is an important regulator of different tiller and leaf angles in grasses, such as rice, wheat, and maize [7,8,9,10,11]. *TAC1* regulates the branch angle of woody plants, such as peach, poplar, and plum [8,12,13]. The mutation of the *TAC1* gene results in a smaller branch angle and an almost vertical upward growth of branches in peach trees [12], while its overexpression results in a larger branch angle and more horizontal branch in plum trees [13]. Tiller angle control 4 (*TAC4*) is a novel regulator of the rice tiller angle. It can regulate rice shoot gravitropism by increasing the indole acetic acid content and affecting the auxin distribution, thereby negatively regulating the tiller angle [14]. Loose plant architecture 1 (*LPA1*) is a homologous gene of the Arabidopsis gravity response gene *AtIDD15/SGR5*, which regulates tiller angle and leaf growth angle by influencing gravity perception and signal transduction [13]. Prostrate Growth 1 (*PROG1*) encodes a single Cys2–His2 zinc-finger protein, and the artificial selection of *PROG1* results in the vertical growth of rice, a heavier grain weight, and a higher yield [15,16]. Bigger tiller angle 1 (*BTA1*) can change the tiller angle by changing the nSegative gravitropic response in rice. Mutations in this gene lead to an increased tiller angle phenotype and changes in agronomic traits such as tiller number, stem length, and effective panicle number [17,18]. The overexpression of the methyl-CpG-binding protein gene *OsMBD707* leads to larger tiller angles and reduced photoperiod sensitivity in rice [19].

The *LAZY* genes are important regulators of lateral organ orientation [20,21]. The *LAZY1* gene plays an important role in plant branch angle. *LAZY1* mutation can lead to an increase in the tiller angle of rice and maize [22,23,24,25,26,27]. The overexpression of *LAZY1* results in a smaller branch angle of poplar trees [6]. *LAZY1* regulates the tiller angle of maize and rice by regulating auxin polar transport and signal transduction [22,25,28]. A loss of *LAZY1* function greatly enhances auxin polar transport, changes the distribution of endogenous IAA in rice seedlings, and increases the tiller angle of rice [22]. There are two gravity response pathways of *LAZY1* in plants: dependent and independent [24]. A large-scale transcriptome analysis of rice stems in response to gravity revealed that *HSFA2D* responds to gravity and induces *LAZY1* transcription, thereby initiating the asymmetric distribution of auxin *WOX6* and *WOX11* expression. These results reveal the core regulatory pathway of tiller angle in rice [27]. In addition, yeast-two-hybrid and BiFC experiments determined that the maize LAZY1 (ZmLAZY1) protein bound to a protein kinase (ZmPKC) and the Aux/IAA auxin signaling repressor protein (ZmIAA17) [25]. In addition to its role in geotropism and stem structure, *LAZY1* is also critical for tassel and ear development in maize [26].

Taken together, although the genes controlling tiller or branch angle have been characterized (mainly in grasses such as rice and corn), the functions and mechanisms regulating the tiller angle of woody plants remain largely unknown. In this study, based on the transcriptome data of ‘Zilong wochi’ rose branches, *RrLAZY1*, which is associated with large differences relating to branch angle, was selected as the target gene and its expression patterns and biological functions were studied, providing valuable insights into the molecular mechanism by which the *RrLAZY1* gene regulates the prostrate habit of rose plants.

## 2. Results

### 2.1. Cloning of RrLAZY1 and Sequence Analysis

**Figure 1:**
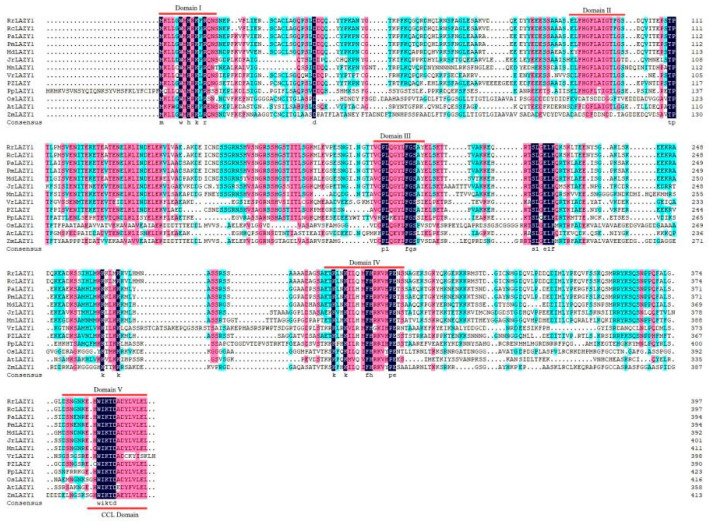
Amino acid sequence homology analysis of *RrLAZY1* and *LAZY1* genes from other species. Five conserved domains of the *LAZY1* gene were presumed to be represented by red lines. The conserved domain II contains the GΦL (A/T) IGT domain. The C-terminus of region *Ⅴ* has a highly conserved CCL domain and contains the EAR motif (L/FVLEL).

**Figure 2:**
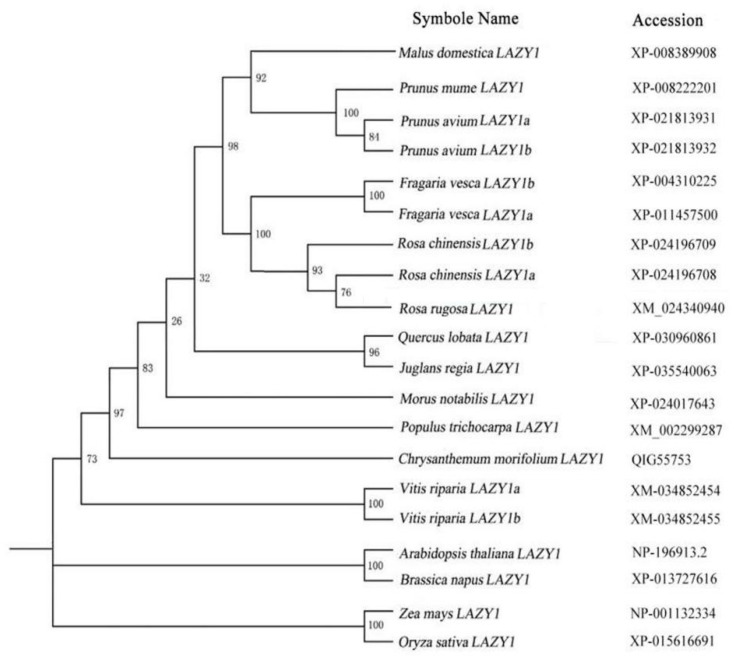
Phylogenetic analysis of *RrLAZY1* and *LAZY1* genes from other species.

The full-length CDS of the *RrLAZY1* gene was obtained with PCR amplification. The coding sequence (CDS) of *RrLAZY1* is 1194 bp in length and is predicted to encode a 397 amino acid (aa) protein, with a molecular weight of 44.17 kDa and an isoelectric point (pI) of 6.17. Multiple amino acid sequences alignment showed that the *RrLAZY1* gene has five conserved regions (Figure 1). In the conserved region II, the *RrLAZY1* gene contains the GΦL (A/T) IGT domain, which belongs to the IGT gene family. The C-terminus of region Ⅴ has a highly conserved CCL domain and contains an EAR motif (L/FVLEL). Additionally, phylogenetic analysis showed that *RrLAZY1* is most closely related to *RcLAZY1* (Figure 2).

### 2.2. Subcellular Localization of RrLAZY1

**Figure 3:**
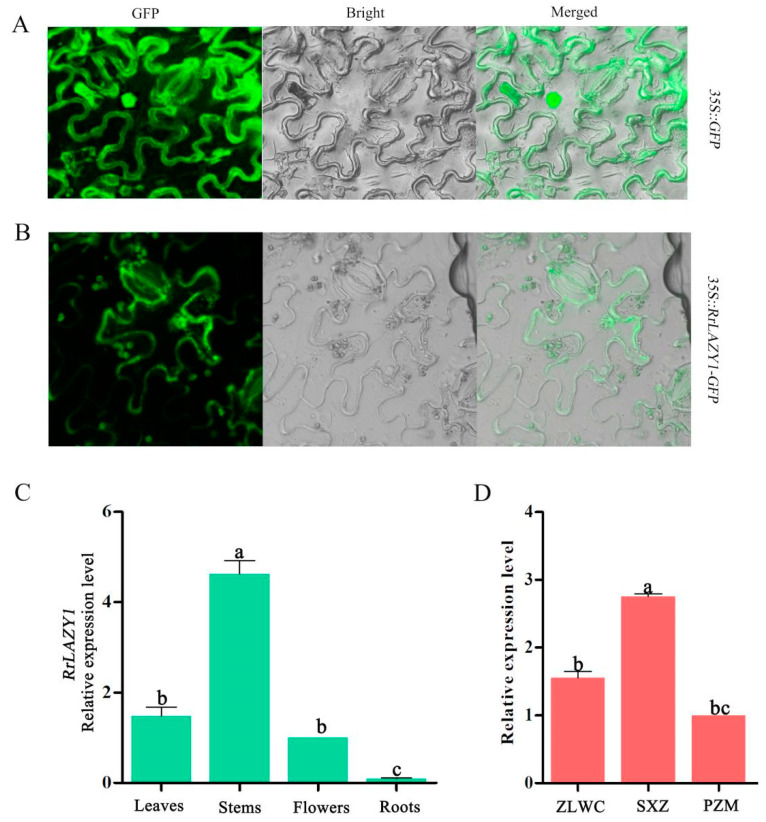
The expression characteristics of *RrLAZY1*. (**A**,**B**) Subcellular localization analysis of the *RrLAZY1* gene. The *35S::RrLAZY1-GFP* fusion vectors were transiently expressed in the epidermal cells of tobacco leaves with 35S::GFP vectors as a control. GFP, GFP fluorescence; Bright, bright field; Merged, superimposition of bright field and fluorescence. (**C**) The relative expression level of the *RrLAZY1* gene in different tissues of *R. rugosa* ‘Zilong wochi’. (**D**) The relative expression level of *RrLAZY1* gene in the stems of *R. rugosa* of two architecture types. SXZ, *R. rugosa* ’Saixizi’; ZLWC, *R. rugosa* ’Zilong wochi’; PZM, *R. rugosa* ’Pingzhimei’. Different lowercase letters indicate significant differences at *p* < 0.05.

The GFP fusion protein was constructed to investigate the subcellular localization of *RrLAZY1*. The results of transient expression analyses showed that the GFP signal of the control (the empty vector) pCAMBIA1302-GFP was localized throughout the entire cell (Figure 3A). However, recombinant *RrLAZY1-GFP* was localized in the cell membrane of tobacco leaves (Figure 3B). These results indicate that *RrLAZY1* is a membrane-localized protein.

### 2.3. Expression Patterns of RrLAZY1 in Rosa

In order to investigate the expression pattern of *RrLAZY1*, total RNAs were extracted from leaves, stems, flowers, and roots. The results of a qRT-PCR showed that the *RrLAZY1* gene was expressed in four different tissues—specifically, it was highly expressed in stems, while exhibiting relatively low expression levels in flowers and roots (Figure 3C). Notably, the expression levels of *RrLAZY1* in the erect variety (SXZ) were significantly higher than those in the two creeping varieties (ZLWC, PZM) (Figure 3D).

### 2.4. Identification of Transgenic Arabidopsis Plants

**Figure 4:**
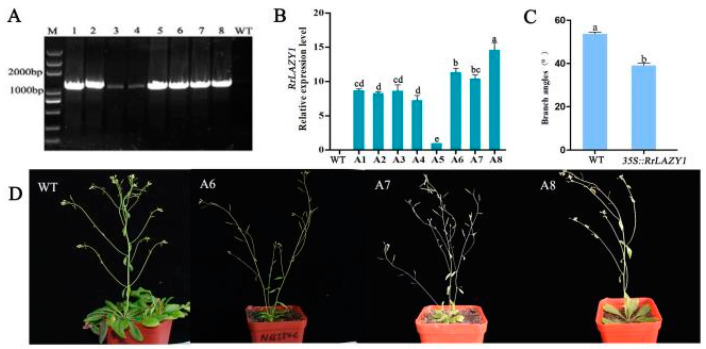
Analysis of Arabidopsis lines overexpressing the *RrLAZY1* gene. (**A**) PCR identification of *RrLAZY1* overexpression and wild-type Arabidopsis plants; (**B**) the results of the qRT-PCR detection of transgenic Arabidopsis lines; (**C**) inflorescence branch angle of transgenic Arabidopsis; Different lowercase letters indicate significant differences at *p* < 0.05. (**D**) phenotypes resulting from the overexpression of *RrLAZY1* in Arabidopsis.

The *35S::RrLAZY1* construct was transformed into the genomes of wild-type Col-0 Arabidopsis plants. Transgenic Arabidopsis lines were generated via the floral dip method. Eight *RrLAZY1* transgenic Arabidopsis lines were obtained by Hyg resistance screening (Figure 4A). qRT-PCR analysis showed that the expression level of *RrLAZY1* was significantly higher in the transgenic plants—especially A6, A7, and A8—than in the plants of the control group (Figure 4B). Therefore, these three lines were used for further experiments.

### 2.5. Phenotypic Observation of Transgenic Arabidopsis Plants

After two successive generations of screening, phenotypic observations and related tests of the T2 generation of transgenic plants were carried out. Compared with wild-type Arabidopsis (WT) plants, the inflorescence of *35S::RrLAZY1* transgenic Arabidopsis showed a state of curved growth with significantly smaller branch angles (Figure 4D). As shown in Figure 4C, the *35S::RrLAZY1* branch angle (39.12°) was significantly smaller than those of the WT (53.71°), indicating that the *RrLAZY1* gene can negatively regulate the branch angle.

**Figure 5:**
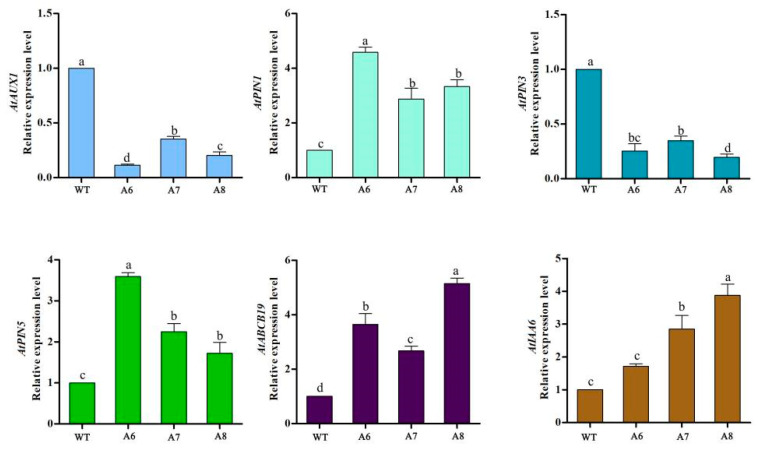
Expression analysis of auxin-related genes in *RrLAZY1* transgenic Arabidopsis lines and the WT. WT, wild-type Arabidopsis. Different lowercase letters indicate significant differences at *p* < 0.05.

By detecting the expression levels of genes related to auxin transport and signal transduction in Arabidopsis plants, it was found that the overexpression of the *RrLAZY1* gene changed the expression levels of auxin transport vectors and genes related to signal transduction in Arabidopsis. Compared with that in the WT, the expression of *AtAUX1* and *AtPIN3* was significantly downregulated, while the expression of *AtPIN1*, *AtPIN5*, *AtABCB19*, and *AtAtIAA16* was significantly upregulated (Figure 5). These results suggested that *RrLAZY1* might induce the asymmetrical distribution of auxin by regulating the expression of auxin transport- and signal transduction-related genes and then regulating the branch angle of Arabidopsis inflorescence.

### 2.6. RrLAZY1 Interacts with Auxin Signaling Proteins

**Figure 6:**
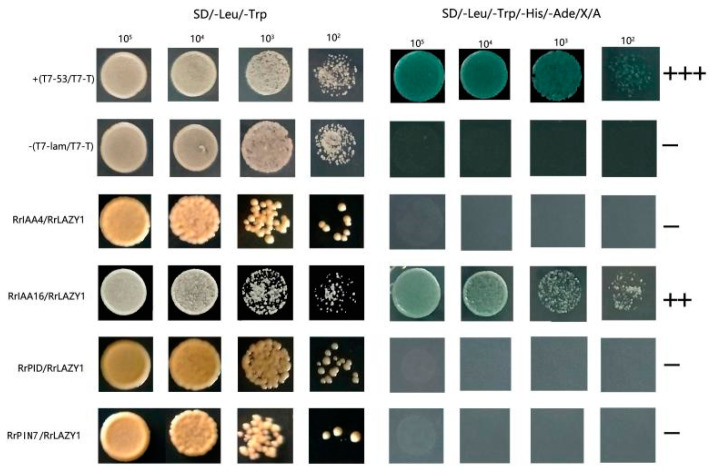
Analysis of protein–protein interactions via yeast two-hybrid experiment between RrLAZY1 and auxin-related proteins of *R. rugosa*. +++, ++, represent very strong and strong interactions, respectively. −, no interaction.

In view of the important role of *RrLAZY1* in auxin polar transport, we screened four genes related to auxin transport or signal transduction in the transcriptome data, including *PIN7*, *PIDOUX (PID), IAA4* and *IAA16*. We performed a yeast two-hybrid assay to screen for LAZY1-interacting proteins. Among several candidate RrLAZY1-interacting proteins, RrIAA16 showed a strong interaction with RrLAZY1 in yeast based on a b-galactosidase activity assay (Figure 6).

**Figure 7:**
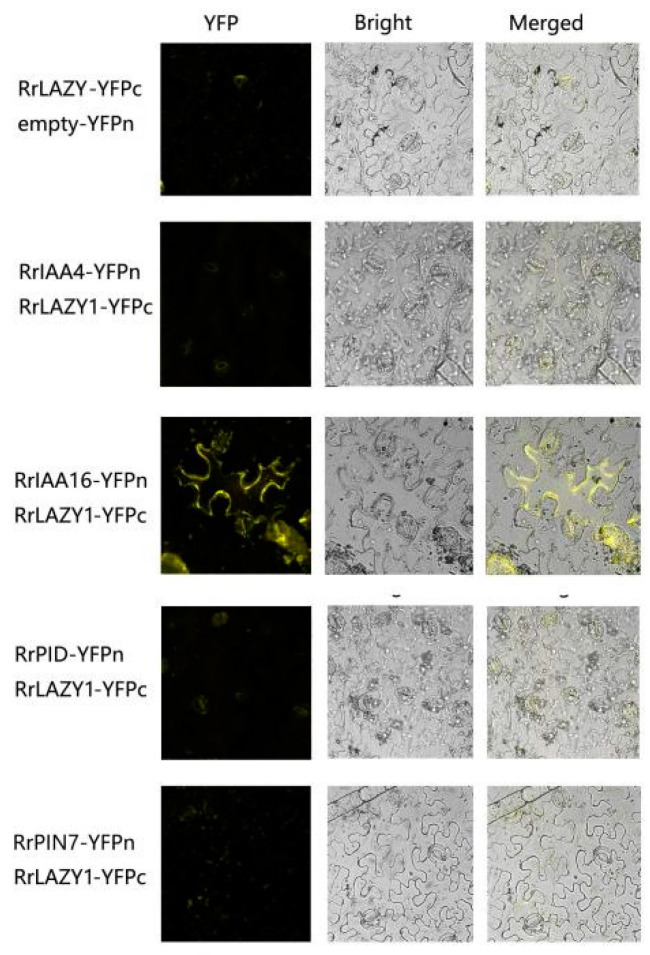
Analysis of protein–protein interactions via bimolecular fluorescence complementation experiments between RrLAZY1 and auxin-related proteins of *R. rugosa*. RrLAZY1-YFPc/RrIAA16-YFPn interact on the cell membrane. There was no yellow fluorescence signal in the control group or other combinations. YFP: YFP fluorescence; Bright, bright field; Merged, superimposition of bright field and fluorescence.

To further verify the interaction of RrLAZY1 with RrIAA16 in plants, we used bimolecular fluorescence complementation experiments to verify the effects of tobacco epidermal cells (Figure 7). The results showed that RrLAZY1 interacted with RrIAA16 on the cell membrane.

## 3. Discussion

Branch angle is an important factor determining the formation of the creeping plant habit in roses. Although several genes related to branch angle have been identified, the regulatory mechanism of branch angle in rose is still unclear. In this study, based on a transcriptome data analysis, we cloned the full-length CDS of the *LAZY1* gene from the branches of rose ‘Zilong wochi’; studied its biological functions, including subcellular localization, tissue-specific expression patterns, protein interactions and identified its functions through heterologous over-expression.

The *LAZY* genes belong to the IGT gene family. These genes encode moderately sized proteins with no known or predictable biochemical functions [24,29,30]. In this study, five conserved regions of the *RrLAZY1* gene were deduced by sequence analysis. The amino acid conservation of regions I and II at the N-terminus and region *V* at the *C*-terminus is relatively strong, while the amino acid conservation in regions III and IV is weak. For Arabidopsis *LAZY1* gene, region I was required for AtLAZY1 proteins to reside at the plasma membrane, which is necessary for its function. Region II contains the GΦL (A/T) IGT motif. Regions III and IV could be mutated without large impact on function or localization. Region V, also named the CCL domain, contains an ethylene-responsive amphiphilic repression (EAR) transcriptional repressor motif. Mutating region V severely disrupts its function without affecting subcellular localization [30]. *RrLAZY1* has a high sequence similarity with *LAZY1* genes of other woody plants, including rose, peach, plum, and poplar [31], suggesting that the *LAZY1* gene is highly conserved in the evolutionary process of woody plants.

Through fluorescence quantitative experiments, we found that the expression levels of *RrLAZY1* were higher in the stem and leaf of rose but lower in the flower and root, which was consistent with the expression patterns of the LAZY1 gene in poplar and sallow [8,32], indicating that the *LAZY1* gene mainly plays a regulatory role in the aboveground part of the plant. Notably, the expression level of *RrLAZY1* was different among different plant types of rose varieties, while the expression level of *RrLAZY1* in the upright plant type ‘Saixizi’ was significantly higher than that in the prostrate plant types ‘Zilong wochi’ and ‘Pingzhimei’, which further indicated that the *LAZY1* gene has a regulatory effect on the branch angle of roses. In addition, the subcellular localization pattern of *RrLAZY1* gene was analyzed, indicating that the RrLAZY1 protein was only localized on the cell membrane, which was consistent with the results of tea plant CsLAZY1 membrane localization [33]. However, *LAZY1* has the characteristics of dual localization of nuclear and cell membranes in maize, rice, and Arabidopsis [22,26,28]. SpsLAZY1a and SpsLAZY1b proteins were only localized in the nucleus of Salix [34]. Therefore, the localization patterns of LAZY1 proteins in cells differ by species.

In this study, the *RrLAZY1* gene was first introduced into Arabidopsis through Agrobacterium-mediated inflorescence impregnation, and corresponding transgenic Arabidopsis lines were obtained. The overexpression of the *RrLAZY1* gene significantly reduced the branch angle of Arabidopsis inflorescences. Therefore, we confirmed that *RrLAZY1* negatively regulates branch angle, which is consistent with the role of *LAZY1* gene over-expression in poplar [8]. *LAZY1* regulates the tiller angle of rice by controlling geotropism and auxin polar transport [15,27]. The *LAZY1* gene is a negative regulator of auxin polar transport and a positive regulator of auxin lateral transport [22]. *LAZY1* gene mutations increase auxin polar transport, alter plant gravity, and ultimately lead to an extreme spreading phenotype in rice [35,36]. Thus, polar auxin transport and asymmetric distribution play key roles in the geotropic response and regulate the branch angle of plants. The overexpression of the *RrLAZY1* gene caused changes in auxin transport- or signal transduction-related genes, which changed the polar transport and distribution patterns of auxin and thus changed the branch angle of Arabidopsis inflorescences.

In addition, the RrLAZY1 protein interacted with the auxin response protein RrIAA16 on the cell membrane. The LxLxL motif contained in the AUX/IAA gene can specifically inhibit the ARF response to auxin signal transduction [37], and there is also a potential EAR motif in the *RrLAZY1* sequence, which can play a negative regulatory role. Therefore, we believe that the *RrLAZY1* gene negatively regulates the branch angle by participating in auxin transport and signal transduction pathways to regulate the expression of auxin-related genes.

## 4. Materials and Methods

### 4.1. Plant Materials

With respect to rose, *R. rugosa* ‘Saixizi’, *R. rugosa* ‘Zilong wochi’ and *R. rugosa* ‘Pingzhimei’ plants cultivated in the Rosa germplasm nursery of Shandong Agricultural University were used as test materials. We collected four different tissue samples (roots, stems, leaves, flowers) on the mornings of sunny days from mid-April to mid-May 2019. After they were flash frozen in liquid nitrogen, all samples, which were collected in triplicate and put into a −80 ℃ refrigerator for storage.

*Arabidopsis thaliana* ‘Columbia’ and *Nicotiana benthamiana* were grown under a 16 h light/8 h dark photoperiod at 23 °C/21 °C.

### 4.2. Total RNA Extraction and cDNA Synthesis

Total RNA was extracted from the stems of the above three rose varieties using an EASY Spin Plant RNA Extraction Kit (Aidlab Biotech, Beijing, China). RNA integrity was analyzed by 1.0% agarose gel electrophoresis. The RNAconcentration and purity were determined by a Nanodrop 2000C ultramicrospectrophotometer (Thermo Fisher Scientific, Wilmington, DE, USA), and the qualified RNA was preserved at −80 °C. First-strand cDNA was synthesized via a 5× All-In-One RT Master Mix Reverse Transcription Kit (ABM Company, Vancouver, Canada) for gene cloning and fluorescence quantitative experiments.

### 4.3. Gene Cloning and Sequence Analysis

The cDNA of the ‘Zilong wochi’ cultivar stems was used as the template. The full-length coding sequence (CDS) of the *RrLAZY1* gene was amplified using PCR. Primers are listed in Supplementary Materials. A phylogenetic tree was constructed using the Evolview program. Sequence homology and alignment were carried out with DNAMAN software (version 7.0, Lynnon Biosoft, Canada).

### 4.4. Subcellular Localization of RrLAZY1

Through the sequence analysis of the *RrLAZY1* gene and the pCAMBIA1302 vector, full-length cDNA without the termination codon of *RrLAZY1* was amplified with special primers with restriction sites (Ncol and SpeI) and subcloned into the pCAMBIA1302-GFP vector to create the *RrLAZY1*-GFP fusion construct. The recombinant vectors and control vector (pCAMBIA1302-GFP) were then introduced into tobacco leaves by agroinfiltration. After 48 h of incubation, the green fluorescence signal was observed and photographed under a laser confocal microscope (LSM880, Zeiss, Germany).

### 4.5. Identification and Phenotypic Observation of RrLAZY1 Transgenic Arabidopsis Plants

The recombinant vector *35::RrLAZY1-pCAMBIA1304* was constructed and transferred into Agrobacterium strain GV3101 by the freeze–thaw method. The recombinant vector was inserted into the wild-type Arabidopsis genome by the inflorescence impregnation method. Arabidopsis-positive transgenic seedlings were screened on medium containing hygromycin and identified by qRT-PCR, and phenotypic changes in the transgenic Arabidopsis strains were observed.

### 4.6. Expression Analysis via Quantitative RT-PCR

Using qRT-PCR, the *RrGAPDH* and *AtActin* genes were used as internal reference genes and the expression levels of the *RrLAZY1* gene and auxin-related genes in *R. rugosa* and *Arabidopsis thaliana* were detected by a CFX-96 real-time quantitative RT-PCR instrument according to the instructions of the SYBR^®^ Premixture Ex Taq^TM^. Each sample included 3 independent biological replicates. The PCR conditions were as follows: 95 °C for 30 s, 40 cycles of 95 °C for 5 s and 60 °C for 30 s and then a dissociation stage at 95 °C for 10 s, 65 °C for 5 s and 95 °C for 5 s. All gene-specific primers used in this study are shown in Supplementary Materials. The relative expression levels of genes were calculated using the 2^−∆∆Ct^ method.

### 4.7. Yeast Two-Hybrid Assays

The full-length sequence of the *RrLAZY1* gene was ligated into the pGBKT7 bait vector, and four auxin-related genes were ligated into the pGADT7 prey vector. The bait plasmid was transferred into the Y2HGold strain according to the manufacturer’s instructions. Aureobasidin A (AbA) was used to screen the minimal inhibitory concentration for the bait strains. The bait and pellets were transferred into the Y2HGold strain by the cotransfer method, and positive colonies were identified by standard colony PCR screening. The transformed yeast cells were diluted with a 10× dilution series and dotted on SD/-Leu/-Trp and SD/-Leu/-Trp/-His/-Ade with an optimal concentration of X-α-Gal and AbA. After culture at 30 °C for 3–5 days, the growth of yeast cells was recorded.

### 4.8. Bimolecular Fluorescence Complementation Experiment

The full-length sequence of the *RrLAZY1* gene was ligated to the pSPYCE (C-terminal) vector, and the four auxin-related genes were ligated to pSPYNE (N-terminal). These recombinant plasmids were transformed into Agrobacterium strains and injected into tobacco leaves. After culturing for 48 h, the yellow fluorescent signal was observed under a laser confocal microscope (LSM880, Zeiss, Germany).

### 4.9. Data Analysis

Three independent biological replicates were measured for each sample, and the data were presented as the mean ± standard error (SE). Where applicable, data were analyzed using a Student’s t test in a two-tailed analysis. Values of *p* < 0.05 were considered statistically significant.

## 5. Conclusions

In the present study, we identified *RrLAZY1* gene from *R.* *rugosa*. The *RrLAZY1* gene showed distinct expression patterns in four different tissues, and had the highest expression level in the stem. The RrLAZY1 protein was localized in the plasma membrane based on a subcellular localization analysis. The over-expression of *RrLAZY1* in Arabidopsis resulted in a smaller branch angle and altered auxin related gene expression levels. Studying and identifying the function of the *RrLAZY1* gene in roses will provide important theoretical support for improving our knowledge of the branching direction and plant structure of roses and provide new ideas for further revealing the molecular mechanism of the formation of creeping rose plants.

## Data Availability

Not applicable.

## Supplementary Materials

The following are available online at https://www.mdpi.com/article/10.3390/ijms222413664/s1.
