# Supplementary material for: Over-Expression of Rose RrLAZY1 Negatively Regulates the Branch Angle of Transgenic Arabidopsis Inflorescence
Source: Int J Mol Sci. 2021 Dec 20;22(24):13664. doi: 10.3390/ijms222413664 (PMC8709306; doi:10.3390/ijms222413664)
Supplement: Supplementary file 1 [file ijms-22-13664-s001.zip › ijms-1477115-supplementary.pdf]

Supplementary material

Table S1. Primer sequences of related genes used in the study.

Gene	Primer name	Sequence (5′-3′)	Description
RrLAZY1	LA-F	ATGAAGTTACTGGGATGGATGC	cDNA full-length amplification primers
	LA-R	TCACAGCTCCAGGACTAGGTAGT	
RrIAA4	IAA4-F	ATGGCTTCCAAGCAGAGG	
	IAA4-R	TCACCCGCACAACCTAAG	
RrPIN7	PIN7-F	ATGGGTTTCTGGACTCTGC	
	PIN7-R	TCAAGACAAGATCCACATGTAG	
RrPID	PID-F	ATGTTAGAGCTTGGTCCGATTAG	
	PID-R	TCAAAAGTAATCGAACGCC	
RrIAA16	IAA16-F	ATGACCAGTACTGCTGCTATGG	
	IAA16-R	TCAACTTCTGTTCTTGAACTTCTCC	
RrLAZY1	LA+S-F	ACTAGTATGAAGTTACTGGGATGGATGC	Primers for over-expression vector construction
	LA+B-R	GGTCACCTCACAGCTCCAGGACTAGGTAGT	
RrLAZY1	QA-F	TCTTGCAGGCCAGCCATCAC	Fluorescence quantitative primers
	QA-R	AGATGCCGCAGCAGAGGATT	
RrGAPDH	GAPDH-F	TGAAGGGTGGTGCCAAGAA	
	GAPDH-R	AAGGGGAGCAAGACAGTTGG	
AtPIN1	QPIN1-F	TCACACCAGACCAATGCTCC	Quantitative PCR primers for Arabidopsis over-expression
	QPIN1-R	TCTAAAGAACCGTTGCGGCT	
AtPIN3	QPIN3-F	GCCTCGAGTGGAGCATCACA	
	QPIN3-R	GCGCCACGAAACTCGAAGAG	
AtPIN5	QPIN5-F	CTATTTCACCCTGCCGCTCT	
	QPIN5-R	CCACAACGCTAAGACCGTGA	
AtAUX1	QAUX1-F	GGAAAGCACTAGGACTCGCA	
	QAUX1-R	TGGCACAAGCAATGAGTTGG	
AtABCB19	QABCB19-F	GGTGCTTACGCATCCCTCAT	
	QABCB19-R	AGTGAATGGCTCAAACGGGT	

AtIAA6	QIAA6-F	GAAGAGTCAAGCGGTGGGAT	
	QIAA6-R	GCCACTCCTATGCCAAGACA	
AtAction	QACT-F	TCTCTATGCCAGTGGTCGTA	
	QACT-R	CCTCAGGACAACGGAATC	
RrIAA4	IAA4+N-F	ACTAGTATGGCTTCCAAGCAGAGG	Primers used in the construction of bimolecular fluorescent complementary vectors
	IAA4+N-R	CTCGAGCCCGCACAACCTAAG	
*Rr*PIN7	PIN7+N-F	ACTAGTATGGGTTTCTGGACTCTGC	
	PIN7+N-R	AGTACTAGACAAGATCCACATGTAG	
RrPID	PID+N-F	GGATCCATGTTAGAGCTTGGTCCGATTAG	
	PID+N-R	CTCGAGAAAGTAATCGAACGCC	
RrIAA16	IAA16+N-F	GGCGCGCCATGACCAGTACTGCTGCTATGG	
	IAA16+N-R	CTCGAGACTTCTGTTCTTGAACTTCTCC	
RrLAZY1	LA+C-F	ACTAGTATGAAGTTACTGGGATGGATGC	
	LA+C-R	CTCGAGCAGCTCCAGGACTAGGTAGT	
RrLAZY1	LA+B-F	GAATTCATGAAGTTACTGGGATGGATGC	Primers constructed by yeast two-hybrid vector
	LA+B-R	GGATCCTCACAGCTCCAGGACTAGGTAGT	
RrIAA4	IAA14+A-F	CATATGATGGCTTCCAAGCAGAGG	
	IAA14+A-R	CTCGAGTCACCCGCACAACCTAAG	
RrPID	PID+A-F	GGATCCATGTTAGAGCTTGGTCCGATTAG	
	PID+A-R	CTCGAGTCAAAAGTAATCGAACGCC	
*Rr*PIN7	PIN7+A-F	CATATATGGGTTTCTGGACTCTGC	
	PIN7+A-R	GGATCCTCAAGACAAGATCCACATGTAG	
*Rr*IAA16	IAA16+A-F	CATATGATGACCAGTACTGCTGCTATGG	
	IAA16+A-R	CTCGAGTCAACTTCTGTTCTTGAACTTCTCC	
RrLAZY1	A+Y-F	CCATGGATGAAGTTACTGGGATGGATGC	Primers for subcellular localization vector construction
	A+Y-R	ACTAGTCAGCTCCAGGACTAGGTAGT	
